# Novel Stentless Strategy With Perfusion and Drug-Coated Balloons for Treating Acute Coronary Syndrome

**DOI:** 10.1016/j.jscai.2023.101175

**Published:** 2023-10-11

**Authors:** Ryota Fukuoka, Tomohiro Kawasaki, Kyoko Umeji, Yoshiya Orita, Hisashi Koga, Keisuke Hirai, Kazuki Haraguchi, Yurie Fukami, Kimihiro Kajiyama, Toshiya Soejiyma

**Affiliations:** Cardiovascular Center, Shin-Koga Hospital, Kurume City, Japan

**Keywords:** gradual long inflation, high bleeding risk, low bailout stent rate, plaque rupture, stent-like result

## Abstract

**Background:**

The challenge with the stentless strategy is that the method of optimal predilatation, and predictors of success remain unknown. Studies involving the stentless strategy prior to predilatation are limited. This study aimed to evaluate the long-term efficacy and safety of a new stentless strategy for treating acute coronary syndrome (ACS) using gradual, prolonged predilation with a perfusion balloon combined with a drug-coated balloon (DCB).

**Methods:**

This was a single-center, prospective, single-arm study. A total of 30 patients with ACS underwent gradual, prolonged predilation using a perfusion balloon for 10 minutes, followed by additional dilation using a DCB. The primary end point was target vessel failure at 24 months. Secondary end points were a composite of acute end points, including stentless strategy success rate, procedural complications, final grade of coronary dissection, acute coronary occlusion, in-hospital major adverse cardiac events, and a chronic end point of target vessel failure at 24 months.

**Results:**

A successful stentless strategy was achieved in 24 patients (80%), and 2 (8.3%) patients required ischemic-driven target lesion revascularization in the chronic phase. Six (20%) patients required stent placement due to type C dissection or acute recoil. No acute occlusion and in-hospital major adverse cardiac events were reported.

**Conclusions:**

A novel stentless strategy using predilation with a perfusion balloon and DCB may be helpful for the revascularization of patients with ACS.

## Highlights

• Stentless treatment of acute coronary syndrome uses gradual, prolonged predilation.
• A perfusion balloon combined with a drug-coated balloon was used.
• The stentless strategy achieved successful revascularization in most patients.
• No acute occlusion or in-hospital major adverse cardiac events were reported.
• A low incidence of target vessel failure at 24 months was reported.

## Central Illustration

**Figure:**
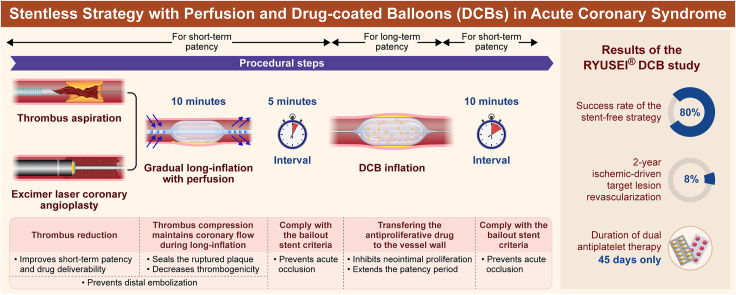

## Introduction

The proportion of patients with high bleeding risk (HBR) is expected to increase due to rapid aging of the population undergoing percutaneous coronary intervention (PCI).^1^^,^^2^ Preliminary evaluation of HBR is often challenging in patients undergoing acute coronary syndrome (ACS) treatment. Postoperative hemorrhagic events are directly linked to prognosis.^3^^,^^4^ Some studies have reported a shortened dual antiplatelet therapy (DAPT) duration due to the use of stentless strategies.^3^^,^^5^^,^^6^ However, many studies on stentless strategies have only included lesions with successful predilatation,^5^, ^6^, ^7^, ^8^, ^9^ as optimal predilatation is essential for a stentless strategy. To date, no study has clarified how to perform predilatation prior to drug-coated balloon (DCB) use. Perfusion balloons are widely used as bailouts for the management of coronary rupture because they allow prolonged dilatation while maintaining coronary blood flow. In percutaneous coronary angioplasty, perfusion balloons have the advantage of allowing lesions to dilate gradually from low pressure, reducing vascular injury and acute recoil with better expansion, compared to conventional balloons.^10^^,^^11^ However, there are no reports of combined DCB and gradual, prolonged dilatation using a perfusion balloon.

This study aimed to clarify the short-term safety and efficacy of gradual, prolonged balloon inflation for 10 minutes using a perfusion balloon for ACS and the long-term effectiveness of its combination with DCB.

## Methods

### Study design and objective

The RYUSEI DCB study was a single-center, prospective, proof-of-concept study. Patients with ACS were enrolled between April 2019 and October 2019.

This study had 2 primary objectives: (1) to evaluate the efficacy and safety of gradual, prolonged balloon inflation using a perfusion balloon (RYUSEI, Kaneka Medical) for lesion preparation prior to DCB use in patients with ACS; and (2) to evaluate the efficacy of treatment regimens regarding the occurrence of target vessel failure (TVF). TVF was defined as cardiac death, recurrent myocardial infarction (MI) in the target vessel, and ischemia-driven target lesion revascularization (TLR) in 24 months postoperatively. This study was performed according to the Declaration of Helsinki guidelines and the study protocols were approved by the institutional review board ethics committee. All patients provided signed informed consent before the procedure. Details regarding the RYUSEI perfusion balloon are provided in the Supplemental Methods.

### Safety concept for applying the perfusion balloon to ACS

According to previous reports, ≥40 mL/min of coronary blood flow is required to prevent myocardial ischemia during the procedure.^12^ Kamioka et^13^ al reported that a coronary driving pressure of ≥60 mm Hg under a pulled-out coronary wire was necessary to maintain coronary flow during dilatation using RYUSEI.

Since the coronary driving pressure is expressed by the aortic pressure (Pa)-coronary venous pressure (Pv) formula, the mean Pa is required to be 60 mm Hg + Pv.^14^^,^^15^ Therefore, the required mean Pa was 80 mm Hg, which was obtained by adding the safety margin of 8 mm Hg to the upper limit of the Pv (normal range, 5-12 mm Hg).

Despite sufficient mean Pa, coronary perfusion pressure may decrease with increased Pv; therefore, patients with elevated Pv, such as in ACS with heart failure, were excluded from the study.

In addition to coronary driving pressure, Kamioka et al^13^ reported that the wire on the perfusion balloon was placed in the coronary artery during balloon dilatation. Consequently, coronary blood flow was reduced by up to 50%. Therefore, it is essential to keep the wire in the appropriate position.

Bailout stent criteria proposed by Uskela et al were strictly enforced. These included recoil ≥30%, dissection ≥Type C, and residual stenosis ≥50%.^3^

### Patient inclusion

Patients diagnosed with ACS who were undergoing emergency coronary angiogram (CAG) were screened. Details of the inclusion and exclusion criteria are reported in Supplemental Methods.

Patients with a new culprit lesion in a native coronary artery with a lesion length <20 mm and reference lumen diameter of 2.0 to 3.5 mm were included. Patients with refractory cardiogenic shock, congestive heart failure (cases requiring ≥3 L oxygen), and malignant arrhythmia were excluded.

### Study procedure

#### Index procedure

Before undergoing the catheterization procedure, patients were administered aspirin (100 mg) and a P2Y12 inhibitor, the standard loading regimen in Japan. Clopidogrel 300 mg or prasugrel 20 mg, and unfractionated heparin (8000 IU) were administered.

Coronary angiogram was performed after intracoronary administration of 100 to 200 mg nitroglycerin. Once a thrombus was confirmed on initial angiography, thrombectomy using thrombus aspiration and/or excimer laser coronary angioplasty was recommended.

Using an imaging device was mandatory, and the operator selected between intravenous ultrasound (IVUS) and optical coherence tomography (OCT).

The size selection of the perfusion balloon was set to 1.0 to 1.1 times the diameter of the reference vessel, determined from the imaging scan. Balloon expansion was initiated at 1 atm every 20 seconds, and the pressure was increased by 1 atm every 20 seconds. There was no upper pressure limit; however, pressure was increased until the desired balloon diameter was obtained. To avoid peripheral ischemia due to balloon expansion, several actions were taken. First, catecholamines were used to maintain a mean Pa of ≥80 mm Hg to maintain perfusion flow. Second, once inflation pressure reached 2 atm, the coronary wire was retracted to the proximal marker. The guiding catheter was removed from the coronary artery ostium to maximize the benefits of the perfusion balloon. Finally, although the required total inflation time of the perfusion balloon was ≥10 minutes, the inflation time for each individual was left to the operator’s discretion.

After a perfusion balloon inflation time of >10 minutes, a 5-minute interval was implemented to ensure sufficient acute gain and to confirm that bailout stent criteria were not breached. DCB (SeQuent Please paclitaxel-coated balloon, Nipro) dilatation was performed as per routine. The DCB balloon size was left to the operator’s discretion; however, a balloon-to-artery ratio of 1.0 to 1.1 was strongly recommended. After DCB dilatation, coronary wire retraction and an observation interval of 10 minutes were recommended to ensure stability of the lesion and flow. In addition to the bailout criteria, bailout stents were permitted in cases of hemodynamic instability.

### Postcatheterization treatment protocol

Patients in the RYUSEI DCB group were administered unfractionated heparin (12,000 units/day) for 24 hours postoperatively. Thereafter, DAPT using aspirin (100 mg/d) and a P2Y12 inhibitor (prasugrel 3.75 mg/d or clopidogrel 75 mg/d) was continued for 30 days. Coronary computed tomography angiography (CCTA) was performed prior to patient discharge to confirm vessel patency.

### End points and clinical follow-up

The primary end point was the TVF at 24 months postoperatively. Secondary end points were efficacy based on composite of acute end points such as stent-free success rate, procedural complications, final dissection grade, acute vessel occlusion, in-hospital major adverse cardiac events, and chronic end point of TVF at 24 months.

Postdischarge, medical follow-up was performed at 6, 12, and 24 months, and imaging (CCTA or CAG) was performed during the 12th and 24th months. DAPT duration and bleeding events were also evaluated. During the observation period, those who did not wish to visit the hospital were evaluated by telephonic interview to record occurrences of blood transfusion, heart failure, or chest pain.

### Imaging analysis

Coronary angiogram, IVUS, and OCT were performed at the initial presentation, after predilatation, and at the end of the procedure. In addition, CCTA was performed before discharge and at the time of each follow-up examination. The evaluation indices for each imaging modality are described in Supplemental Table S1.

### Statistical analysis

Continuous variables of a normal distribution are presented as mean ± SD or median values (with IQR) if not normally distributed. Categorical variables are presented as proportions and percentages. To evaluate the procedure’s success, univariate chi-square analyses of the degree of calcification, thrombus-related parameters (thrombus volume, properties, and length), and presence of plaque rupture were performed. A *P* value of ≤.05 was considered statistically significant. Statistical comparisons between continuous or qualitative variables were performed using JMP version 10.0 (SAS Institute Inc).

## Results

**Figure 1:**
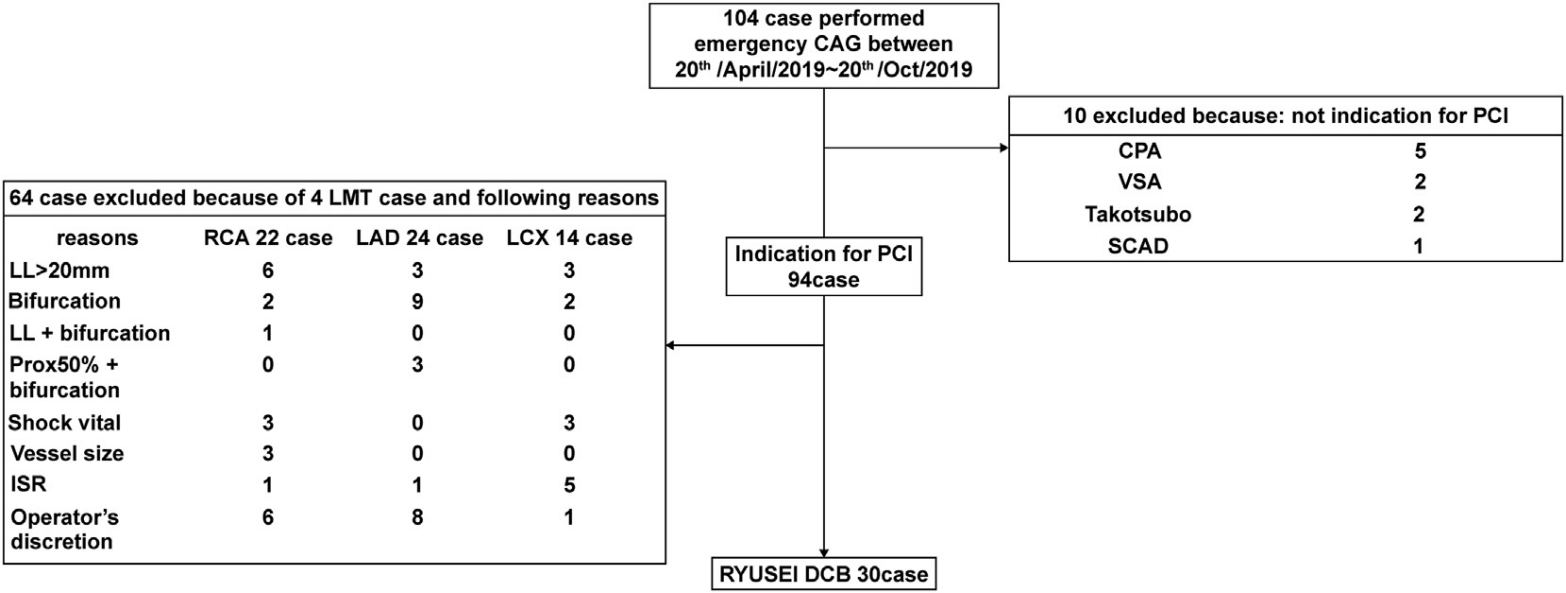
**Flow chart of the study.** CAG, coronary angiogram; CPA, cardiac pulmonary arrest; DCB, drug-coated balloon; ISR, in-stent restenosis; LAD, left anterior descending artery; LCX, left circumflex artery; LL, lesion length; LMT, left main trunk; PCI, percutaneous coronary intervention; RCA, right coronary artery; SCAD, spontaneous coronary artery dissection; VSA, vasospastic angina.

Between April 20, 2019, and October 20, 2019, 30 eligible patients, out of 104 patients who underwent emergency CAG, were assigned to undergo the RYUSEI DCB strategy (Figure 1). Seventy-four patients were excluded, predominantly due to lesion length, bifurcation, combination of lesions, and operator’s discretion.

### Patient characteristics

Thirty consecutive patients with ACS (mean age, 69 ± 13 years; male, 80%, n = 24) were eligible for primary PCI and were treated using the RYUSEI DCB strategy. Of these, 27 (90%) had an acute myocardial infarction (AMI), while 3 had unstable angina (Table 1).

**Table 1:** Basic characteristics of the participants

Characteristic	Value
Presentation	
Acute myocardial infarction	27/30 (90)
Unstable angina pectoris	3/30 (10)
Age, y	69.1 ± 13.0
Male sex	24/30 (80)
Comorbidity	
Hypertension	19/30 (63.3)
Diabetes mellitus	10/30 (33.3)
Dyslipidemia	14/30 (46.7)
Hemodialysis	1/30 (3.3)
Prior myocardial infarction	1/30 (3.3)
Atrial fibrillation	2/30 (6.6)
Smoking, current and past smokers	15/30 (50)
Blood sample, unit	
Hb, g/dL	14.2 ± 1.6
eGFR, mL/min/1.73 m^2^	66.3 ± 21.5
LDL, mg/dL	135.6 ± 38.1
BNP, pg/mL	67 (11.5-171)
CPK, IU/L	167 (91.3-308.8)
Troponin I, pg/mL	640 (78-3533)
DTB (AMI only), min	41 (25-80)
EF by TTE in the emergency room, %	51.2 ± 10.1
Hemodynamics in catheterization laboratory	
Systolic blood pressure, mm Hg	150 ± 29
Mean aortic pressure, mm Hg	107 ± 21.3
SpO_2_, %	98.7 ± 1.2
Loading DAPT	
Aspirin 100 mg	2
Aspirin 100 mg + prasugrel 20 mg	28

Data are expressed as n/N (%), mean ± SD, or median (25th-75th interquartile range).

BNP, brain natriuretic peptide; CPK, creatine phosphokinase; DTB, door to balloon time; EF, ejection fraction; eGFR, estimated glomerular filtration rate; Hb, hemoglobin; LDL, low density lipoprotein; sCr, serum creatinine; TTE, transthoracic echocardiography.

Blood samples obtained at admission demonstrated low brain natriuretic peptide (BNP) levels (median: 67 pg/mL [IQR, 11.5-171]), mild creatine phosphokinase elevation (median 167 IU/L [IQR, 91.3-308.8]), and Troponin I elevation (median 640 pg/mL [IQR, 78-3533]).

Fourteen patients (47%) belonged to the academic research consortium high-risk bleeding group. The median door-to-balloon time in patients with AMI was 41 minutes (IQR, 25-80). The ejection fraction on emergency room transthoracic echocardiography was 51.2 ± 10.1%.

### Baseline characteristics of target lesions

The right coronary artery was the predominant target vessel (15 vessels: 50%). The remaining two-thirds of the lesions (10 lesions) were in the left anterior descending artery. The reference vessel diameter, lesion length, and % stenosis on quantitative coronary angiography of the initial CAG were 2.9 ± 0.5 mm, 19.1 ± 2.6 mm, and 87.5% (IQR, 75-100), respectively (Table 2).

**Table 2:** Baseline characteristics of the target lesions.

Characteristic	Value
Target lesion	
Right coronary artery	15
Left anterior descending artery	10
Left circumflex artery	5
Diagonal branch	1
PD or PL	5
Single vessel disease	17/30 (57)
Initial TIMI flow grade	
0	14/30 (46.7)
1	3/30 (10)
2	7/30 (23.3)
3	6/30 (20)
Rentrope grading	
0	16/30 (53.3)
1	7/30 (23.3)
2	5/30 (16.7)
3	2/30 (6.7)
Preprocedure QCA (all patients)	
Reference diameter, mm	2.9 ± 0.5
Lesion length, mm	19.1 ± 2.6
Minimum lumen diameter, mm	0.34 (0-0.72)
Lesion stenosis, %	87.5 (75-100)
Postprocedure QCA (only stentless strategy)	
Reference diameter, mm	2.9 ± 0.6
Lesion length, mm	19.7 ± 2.5
Minimum lumen diameter, mm	2.1 ± 0.4
Lesion stenosis, %	32 (20-37)

The data are expressed as n (%), mean ± SD, or median (25^th^ to 75^th^ interquartile range).

PD, posterior descending branch; PL, posterolateral artery; QCA, quantitative coronary angiography; TIMI, Thrombolysis in Myocardial Infarction; TTE, thoracic echocardiography.

The baseline thrombolysis in myocardial infarction flow grade was 0/1: 14 (46.7%)/3 (10%) and 2/3: 7 (23.3%)/6 (20%). According to the Rentrope classification for collateral flow, 16 (53.3%), 7 (23.3%), 5 (16.7%), and 2 (6.7%) patients had grades 0, 1, 2, and 3, respectively.

### Procedure characteristics

A thrombus management device was used in 50% of patients (aspiration catheter: 13 cases [43%] and excimer laser coronary angioplasty: 2 cases [6.7%]). No patients had their procedure completed with an aspiration device alone. An imaging device was used for every patient (IVUS: 17 cases [56.6%] and OCT: 13 cases [43.3%]) (Table 3).

**Table 3:** Procedure characteristics.

Characteristic	Value
Inotropic agents	10/30 (33)
Thrombus management device use	15/30 (50)
Aspiration	13/30 (43)
Excimer laser coronary angioplasty	2/30 (6.7)
Guide-extension catheter	8/30 (27)
RYUSEI	
Lesion pass	29/30 (96.7)
Size 2.5/3.0/3.5 mm	8/13/9
Max inflation pressure, atm	11.0 ± 4.5
Max inflation time, min	8.3 ± 2.3
Total inflation time, min	15.5 ± 6.3
Drug coated balloon	
2.5/2.75/3.0/3.5 mm	6/1/10/8
Max inflation pressure, atm	9.8 ± 3.3
Total inflation time, s	60 (57.5-100)
Imaging device use	30/30 (100)
Intravascular imaging	17/30 (56.6)
Optical coherence tomography	13/30 (43.3)
Procedure time, min	95.1 ± 21
Exposure, mGy	1639 ± 726
Contrast volume, mL	182 ± 38.8

The data are expressed as n (%), mean ± SD, or median (25th to 75th interquartile range).

The RYUSEI failed to pass the lesion in 1 patient. A RYUSEI balloon diameter over 3.0 mm was selected in 73% of patients. Dilatation pressure, maximum dilatation time, and total dilatation time were 11.0 ± 4.5 atm, 8.3 ± 2.3 minutes, and 15.5 ± 6.3 minutes, respectively. DCB balloon diameter over 3.0 mm was selected in 72% of patients. The mean total procedural time was 95.1 ± 21 minutes. There was no significant difference in the mean total procedural time between the bailout stent and RYUSEI DCB groups (102 ± 22.8 min vs 93.3 ± 20.5 min, *P* = .37).

### Complications and procedure outcomes

Malignant arrhythmias were not observed during long inflation; however, ventricular fibrillation after reperfusion was observed in 1 case (Table 4). Slow flow, quickly resolved using vasodilators, occurred in 20% of cases after DCB dilatation and was not observed after RYUSEI dilatation. No reflow was not observed in any case. The use of aspiration devices and underlying disease (ST-segment elevation myocardial infarction [STEMI] vs non-STEMI [NSTEMI]) were evaluated as factors involved in slow flow. No significant differences were found between STEMI vs NSTEMI (*P* = .2) or the use of aspiration (*P* = .06).

**Table 4:** Complications and procedure outcomes.

	Details
Complications	
Malignant arrhythmia	1^a^
Acute occlusion	0
No reflow	0
Slow flow	6/30 (20)
Procedure outcomes	
Final dissection classification	
None	13
A	8
B	7
C	1
D/E/F	0
Stent-free rate	24/30 (80)

Data are expressed as n or n/N (%).

a One case of ventricular fibrillation.

The final dissection classification and bailout stent rate were evaluated to assess device efficacy. There was only 1 case of type C dissection. Most cases had no dissection or only a minor dissection (non: 13, A: 8, and B: 7). Bailout stenting was performed in 6/30 (20%) in the intent-to-treat analysis, and 5/29 (17.2%) patients, respectively, excluding 1 case of RYUSEI non-passage.

### Imaging evaluation

#### IVUS findings

The bailout stent group demonstrated no difference in the external elastic membrane; however, it exhibited a larger plaque volume and higher calcification than the RYUSEI DCB group. In addition, deep calcification was more common in the bailout stent group (Supplemental Table S2).

### OCT findings

#### Predictor of optimal predilatation by OCT

Thrombus scores, including thrombus volume and burden (mean thrombus area/mean lumen area), were more extensively observed in the bailout stent group but were not significantly different in the OCT arm (Table 5).

**Table 5:** Optical coherence tomography predictor of optimal predilatation.

	Bailout stent (n = 3)	RYUSEI DCB (n = 10)	*P* value
Thrombus score	40.5 ± 3.5	36.1 ± 19.9	.74
Thrombus length, mm	6.2 ± 0.3	5.21 ± 3.4	.67
Thrombus burden, %	56.3 ± 10.5	54.3 ± 21.8	.89
Thrombus volume, mm^3^	10.2 (9.5-10.9)	12.9 (5.0-25.6)	.49
Thrombus type			.13
White > red	2	2	
White < red	1	8	
Plaque rupture			
Initial or after POBA	0	8/10 (80)	.02
Calcium score	4	1.7 ± 1.2	.02
Thickness, μm	1073 ± 318	566 ± 231	.01
Angle, °	246 ± 42	135 ± 75	.02
Length, mm	12.5 ± 2.4	8.4 ± 4.7	.12

The data are expressed as n (%), mean ± SD, or median (25th to 75th interquartile range).

POBA, plain old balloon angioplasty**.**

Optical coherence tomography showed that softer plaques, where plaque rupture was identified initially or after percutaneous old balloon angioplasty, were significantly more frequent in the stentless strategy group with success (*P* = .02).

In contrast, calcified plaques with higher calcification angles, maximum thickness, and calcification scores were significantly higher in the bail-out stent group (*P* < .05).

**Figure 2:**
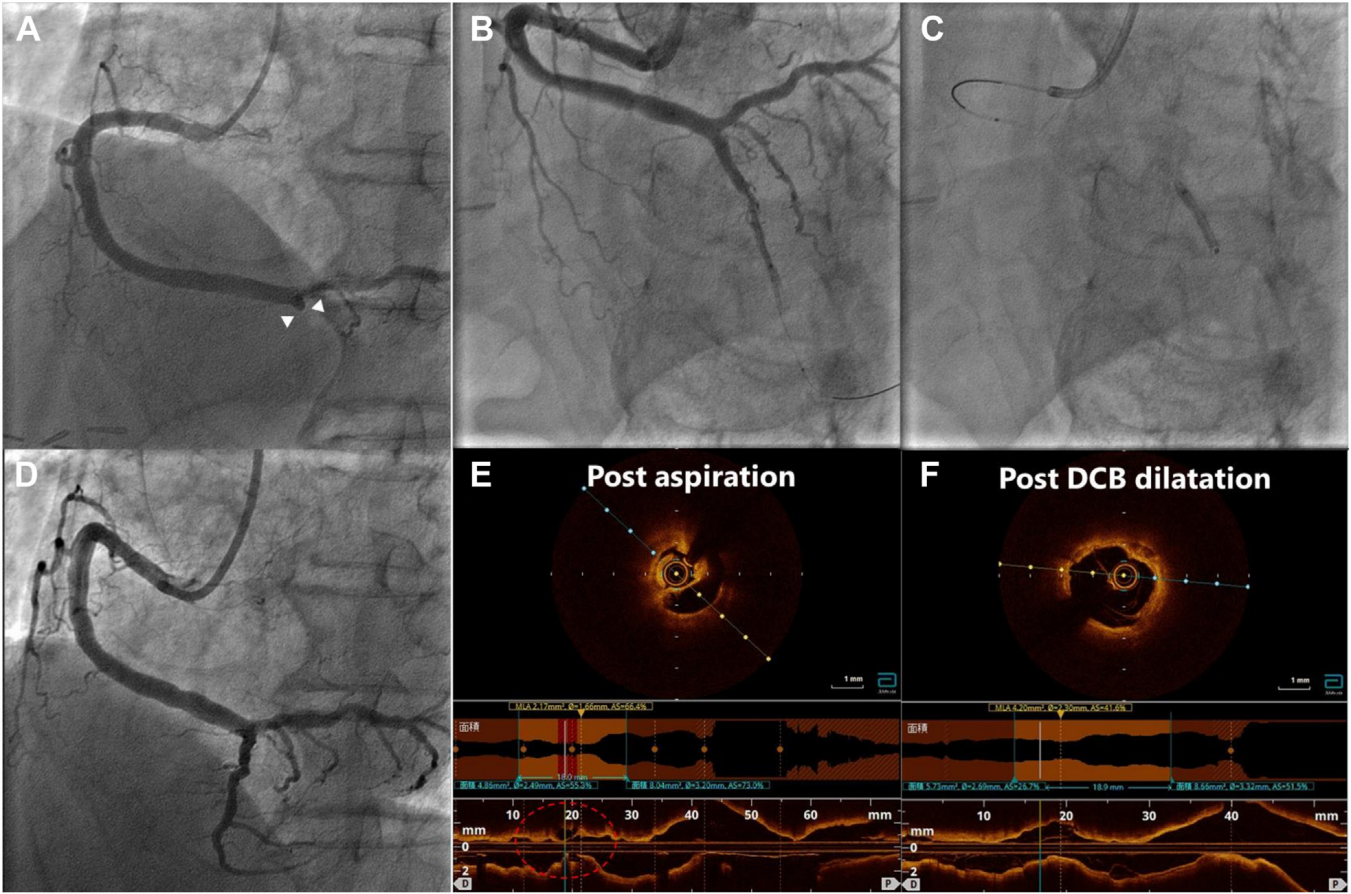
**Typical example of RYUSEI drug-coated balloon (DCB) treatment.** (**A**) Initial coronary angiogram of a patient in his 80s with an ST-segment elevation myocardial infarction; angiogram demonstrates seg 4 posterior descending branch occlusion (arrowhead △). (**B**) After thrombus aspiration, the angiogram demonstrates vessel wall irregularity and ulceration. (**C**) Predilatation, angiogram demonstrates a 3.0 mm RYUSEI in gradual long inflation. (**D**) Final angiogram after DCB dilatation demonstrates a stent-like result. (**E**) Optical coherence tomography (OCT) reveals a large plaque rupture at the culprit lesion (red circle). (**F**) Final OCT reveals a cavity created by the plaque rupture that is well-crimped to the vessel with good acute luminal gain.

Figure 2 demonstrates a typical case of a successful RYUSEI DCB strategy with plaque rupture.

### Short-term outcome after the procedure

No acute occlusion occurred during postoperative hospitalization and CCTA confirmed lesion patency before discharge. Blood samples obtained during hospitalization demonstrated moderate creatine phosphokinase elevation (median, 1340 IU/L [IQR, 234-2403]). CCTA was performed in all patients before discharge to confirm the patency of the treated lesion.

### Long-term outcome

At the 1-year follow-up, all patients were available for telephonic interviews regarding any event occurrence or imaging evaluation. However, 2 did not undergo imaging: 1 had chronic kidney disease, and the other refused to visit the hospital.

In both the RYUSEI DCB and bailout stent groups, 85% were evaluated with CCTA and 15% with CAG.

At the 2-year follow-up, all patients were available for telephonic interviews regarding any event occurrence or imaging evaluation. However, 8 patients did not undergo imaging: 1 had chronic kidney disease, 6 refused to visit, and 1 had a noncardiac death. CCTA or CAG evaluations were performed in 76% of total patients.

**Figure 3:**
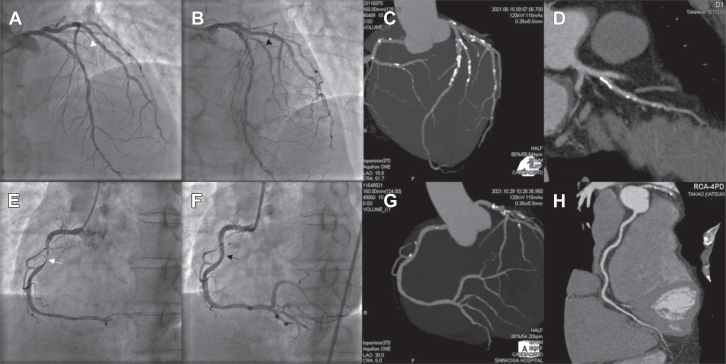
**Typical example of the 2-year follow-up of RYUSEI drug-coated balloon (DCB) treatment**. (**A**) Initial coronary angiogram of a man in his 60s with ST-segment elevation myocardial infarction (STEMI); angiogram demonstrates 1st diagonal occlusion (arrowhead △). (**B**) Final angiogram after DCB (2.5 mm) dilation demonstrates a stent-like result (arrowhead ▲). (**C**) and (**D**) Coronary computed tomography angiography (CCTA) at the 2-year follow-up reveals mild stenosis at the culprit lesion. (**E**) Initial coronary angiogram of a man in his 40s with (STEMI); angiogram demonstrates proximal right coronary artery severe stenosis (white arrow). (**F**) Final angiogram after DCB (3.5 mm) dilation demonstrates a stent-like result (black arrow). (**G**) and (**H**) CCTA at the 2-year follow-up reveals mild stenosis at the culprit lesion.

Figure 3 demonstrates typical cases of CTA assessment at the 2-year follow-up. The DAPT period was a median of 45 (IQR, 33-59) and 190 (IQR, 149-261) days in the RYUSEI DCB and bailout stent groups, respectively.

In the RYUSEI DCB group, 1 patient in their 90s developed bladder and cerebral hemorrhage during single antiplatelet therapy. In contrast, no hemorrhagic event occurred in the bailout stent group.

Target lesion restenosis was observed in 5 (20%) patients in the RYUSEI DCB group and in 1 (16%) patient in the bailout stent group (Supplemental Table S3 demonstrates the coronary CTA findings). Factors involved in target lesion restenosis were evaluated, including the use of aspiration devices and underlying disease (STEMI vs NSTEMI). No significant differences were found between the use of aspiration (*P* = .79) or STEMI vs NSTEMI (*P* = .13).

Regarding TVF, 2 cases had ischemic-driven TLR in the RYUSEI DCB group and 1 in the bailout stent group. No cardiac death or nonfatal myocardial infarction was observed in either group.

All cases with TVF were highly calcified, and the total Agatston score was significantly higher than in the non-TLR group (median 1910 [IQR, 806-3015] vs 152 [IQR, 25.5-36]).

## Discussion

We present an alternative strategy to seal the infarct-related lesion rather than immediate stent implantation in patients with ACS undergoing primary PCI (Central Illustration). The current stentless treatment has presented 3 problems. First, the appropriate method for optimal dilatation is unknown. Second, there are no preceding predictors of success for the stentless strategy. Third, the long-term outcome is unknown. Predilatation of the lesion causing ACS using RYUSEI is a safe and less invasive method for vessel dilatation by gradual prolonged long inflation, with a high procedural success rate of 80%.

**Central Illustration:**
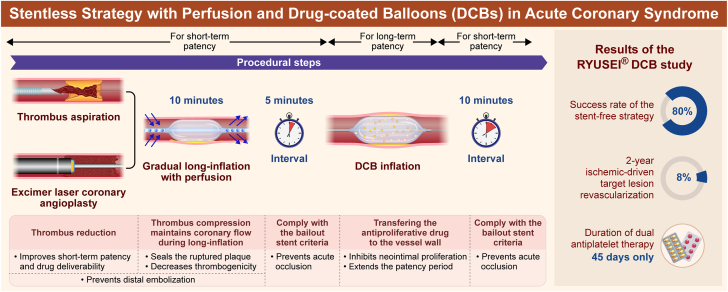
Concept, procedural steps, and results of the RYUSEI DCB study. DCB, drug-coated balloon.

### Effectiveness of RYUSEI with gradual and prolonged dilatation

In the PAclitaxel-eluting balloon in Primary Percutaneous coronary intervention in Amsterdam (PAPPA) pilot study, predilatation of patients with AMI using conventional balloons and DCBs resulted in a >40% bailout stent rate. However, RYUSEI, with gradual, prolonged dilatation in this study, successfully halved the bailout stent rate. In the Revelation trial, which only included patients with successful predilatation, the bailout stent rate after DCB dilatation was similar (18%).^9^

After implementing the stentless strategy in ACS using RYUSEI and a 3-minute long inflation period, Yoshikawa et al^16^ reported a success rate of 24%, which contradicted the results of this study. This discrepancy may have been due to the target lesion differences and dilatation method selection.

We used a large balloon with a balloon-to-artery ratio of 1.0 to 1.1 times, whereas previous reports of DCB stentless PCI used ratios of 0.8 to 1.0 times.^3^^,^^6^^,^^7^ However, the bailout stent rate and degree of suppression of final dissection demonstrated the minimally invasive nature of the gradual prolonged dilatation technique using RYUSEI. This gradual prolonged dilatation method using RYUSEI is not new and was based on the method reported by Ohman et al^10^^,^^11^ in the 1990s and 2000s, before and after the appearance of the bare-metal stent. They reported that gradual, prolonged dilatation with a perfusion balloon resulted in less dissection than conventional balloon dilatation with the same acute gain as a bare-metal stent.

### Predictors of success for stentless strategies

An essential finding of this study is that the presence of plaque rupture on OCT and low calcification score were suggested as predictors of procedural success in stentless PCI.

As no previous reports have demonstrated predictors of success of the stentless strategy, only cases with successful predilatation were included. However, we believe that performing the procedure based on the above lesion characteristics will improve the procedure’s success.

Lesions containing plaque rupture are usually soft, have a high plaque burden and high thrombus volume, and are at risk for distal embolism, stent protrusion, and intraprocedural stent thrombosis at stent placement.^17^

The less invasive RYUSEI with gradual, prolonged dilatation can dilate the lesion without inducing further thrombogenicity, causing rupture, or releasing tissue factor or other potent thromboprophylactic agents from the plaque. In addition, comparing pretreatment and posttreatment OCT images, the cavity caused by plaque rupture was repaired by gradual and prolonged dilatation (Figure 2).

### Long-term outcome

There were 2 ischemic-driven TLR in the RYUSEI DCB group and 1 in the bailout stent group at the 2-year follow-up. One case of early restenosis involved a dialysis patient who developed unstable angina pectoris due to severe stenosis of the right coronary artery ostium with calcified nodules as at primary treatment (total Agatston score of 6273 and target vessel of 3015 on CCTA at discharge). The patient underwent an orbital atherectomy device and drug-eluting stent (DES) implantation in a short period (4 months); however, the patient also required additional drug-eluting stent implantation due to recurrent stenosis 6 months later.

Another patient with TLR presented with AMI with a sandwich-like highly calcified lesion in the proximal left anterior descending artery of the culprit lesion (CCTA at discharge with a total Agatston score of 1661 and target vessel of 805). CCTA at the 2-year follow-up demonstrated a silent occlusion.

Although the ischemic-driven TLR in this study was higher than that in the PAPPA and Revelation trials, previous reports initially excluded heavy calcification.

In contrast, this study’s mean calcium score on CCTA was 370 (mean total Agatston score). Therefore, long-term results were favorable, considering the differences in the inclusion criteria of patients.

The performance of DCB for ACS has been criticized for the higher risk of restenosis due to the inhibitory effect of thrombi on paclitaxel; however, in this study and previous studies on stentless strategy, TLR was comparable to DES.^3^^,^^18^

Pathologically, Nakazawa et al^19^ reported on the affinity of paclitaxel to the lipid-rich plaque (ie, necrotic core) and that it strongly inhibited neointimal proliferation. Generally, lesions with plaque rupture are rich in lipid components and mixed with thrombus, with poor DES performance in the acute and chronic phases.^17^^,^^20^^,^^21^

Despite the small sample size of this study, the success of gradual, long-duration dilatation using RYUSEI was predicted by the presence of vulnerable plaque with plaque rupture. The combination of antiproliferative drugs and gradual prolonged dilatation with RYUSEI resulted in good acute and chronic effects without perioperative complications.

The findings of this study can be used to provide a true imaging-guided PCI. The RYUSEI DCB strategy should be considered first in cases of plaque with PR and backward attenuation on OCT or IVUS for fewer complications.

### Limitation

Several limitations should be acknowledged. First, this study was conducted in a single-center, single-arm setting with a small sample size because the clinical trial was still verifying the feasibility and safety of the new strategy of gradual prolonged dilatation of RYUSEI for ACS. The safety of gradual prolonged dilatation of RYUSEI for ACS was subsequently confirmed. Depending on the patient’s background, PCI may be performed in an unexpected state of increased thrombogenicity. The safety and efficacy of RYUSEI gradual prolonged dilation for ACS requires further study in a larger number of patients, but maintenance of blood flow during RYUSEI dilation and adherence to bailout stent criteria with sufficient time after balloon dilatation can safely provide protection. Second, patients with shock or heart failure were excluded from the study to maintain a high coronary perfusion pressure. In addition, the lesion length was limited to ≤20 mm with respect to the RYUSEI length. Therefore, our results cannot be extrapolated to all patients with ACS. Third, there was a reduced frequency of imaging evaluations, including CT and CAG, conducted during the 2-year follow-up period. However, the presence or absence of events, including chest pain and heart failure, was confirmed in all patients by telephone interview. Furthermore, 12-month follow-up imaging evaluations were available for all except 2 cases, comparable to other studies.

## Conclusion

The new stentless strategy for ACS using RYUSEI for predilatation is a promising short- and long-term method. This new strategy is very effective for patients with HBR and high-risk lesions in low perioperative complications and a short DAPT period. A future larger randomized controlled trial is warranted to evaluate the efficacy and safety of this strategy compared to the current standard of care.
