# Supplementary material for: Novel Stentless Strategy With Perfusion and Drug-Coated Balloons for Treating Acute Coronary Syndrome
Source: J Soc Cardiovasc Angiogr Interv. 2023 Oct 11;3(1):101175. doi: 10.1016/j.jscai.2023.101175 (PMC11307623; doi:10.1016/j.jscai.2023.101175)
Supplement: Supplemental Tables S1-S3 [file mmc1.docx]

**RYUSEI DCB Study**

Supporting Information

1. Supplementary Methods
2. Supplementary Tables (Table S1 and Table S2)

**Supplementary Methods**

1. **Exclusion criteria**

Patients with a history of: 1) cardiogenic shock requiring treatment with inotropes, pressure elevators, or mechanical left ventricular assistance; 2) heart failure requiring respiratory assistance with 3 L or more oxygen; 3) malignant arrhythmia; and 4) contrast media allergy were excluded.

Lesion exhibiting the following characteristics: 1) stenosis of ≥50% proximal to the target lesion; or 2) side branches of ≥2.0 mm were also excluded.

1. **Definition of acute coronary syndrome (ACS):**

ACS is defined as ST-elevation with chest pain, new persistent ST-segment elevation, cardiac troponin T rise and fall, and new regional wall motion abnormalities. Patients with non-ST-elevation acute coronary syndrome with at least two episodes of angina at rest, or one episode lasting 20 min during the preceding 48 h with normal troponin T levels (unstable angina) or rise and fall of high-sensitivity troponin T levels (NSTEMI) are also considered as having acute coronary syndrome.

1. **Definitions of target lesion failure (TLF)**

Target lesion failure (TLF) was defined as the composite of cardiac death, recurrent myocardial infarction, and ischemia-driven target lesion revascularization (TLR).

Cardiac death was defined as death in the presence of acute coronary syndrome, significant cardiac arrhythmia, or refractory congestive heart failure or death attributed to cardiovascular cause at post-mortem.

Recurrent myocardial infarction was defined as characteristic chest pain accompanied by a rise of more than twice the upper reference limit of troponins, the development of new Q waves on the ECG, or both.

Ischemia-driven target lesion revascularization was defined as either percutaneous or surgical revascularization at the culprit lesion site identified at the index procedure for angina or angina equivalent symptoms.

Major bleeding was defined as any fatal bleed, intracranial hemorrhage, pericardial hemorrhage with cardiac tamponade, hypovolemic shock/severe hypotension caused by bleeding requiring pressor or surgery; a fall in hemoglobin ≥5 g/dL; or a need for transfusion of ≥4 units red cell concentrates. Major bleeding was also defined as significantly disabling bleeding, a fall in hemoglobin of ≥3 g/dL but <5 g/dL, or a need for transfusion of at least two units of red cells.

1. **Details of perfusion balloon “RYUSEI®**

For more information about RYUSEI®, see RYUSEI® product Information in KANEKA MEDIX CORPORATION.

RYUSEI® perfusion balloon is similar to the previous perfusion balloon that is compatible with 6 Fr guiding catheters and has inlets and outlets at both ends to maintain coronary blood flow during balloon expansion.

RYUSEI® perfusion balloon has a low-profile tip that improves the passage of a catheter compared to past models. In addition, the catheter has 16 proximal perfusion holes (φ300) from the proximal to distal balloon segment.

1. **Coronary angiogram analysis**

A quantitative coronary angiography (QCA) analysis was performed according to a previously established standard procedure using the Cardiovascular Angiography Analysis System (CAAS 5.10, Pie Medical Imaging B.V., Maastricht, the Netherlands). Coronary flow was assessed with the thrombolysis in myocardial infarction (TIMI) flow grade classification. Reference vessel diameter (RVD), minimal lumen diameter (MLD), diameter stenosis (DS), and lesion length were measured pre-procedure (initial), post-procedure (baseline), and at follow-up. If >50% of the stenosis was found in QCA evaluation, it was considered restenosis. TLR was defined as any repeat percutaneous or surgical intervention due to restenosis in the treated segment. TLR was clinically indicated in cases of restenosis > 50% by QCA, associated with recurrent angina or objective signs of silent ischemia (stress tests or fractional flow reserve), and in case of restenosis ≥70% by QCA without the aforementioned signs or symptoms.

1. **IVUS and OCT image analysis**

Geometric quantitative IVUS and OCT analyses were performed according to the IVUS and OCT clinical expert consensus documents, with routine subsampling; every 1 mm for IVUS analysis and every 0.4 mm for OCT analysis.^1,2,3^

The IVUS and OCT images were evaluated by two cardiologists blinded to the procedural background.

Intracoronary nitroglycerin 200 μg was administered routinely before the image acquisition at baseline and after DCB dilatation. The characteristics of imaging devices and evaluation indices are described in Table S1.

For IVUS data acquisition, motorized transducer pullback system (3.0 mm/s) and a 3.0-Fr IVUS imaging catheter (Alta View, Terumo) incorporated with a 60-MHz phased-array transducer were selected.

The OCT was performed using a commercially available system (OPTIS Mobile systems; Abbott, Santa Clara, CA, USA) and a rapid exchange catheter (Dragonfly™ OPTIS™; Abbott, Santa Clara, CA, USA) with an integrated pullback system (18-36 mm/s).

In IVUS analysis, external elastic membrane (EEM), lumen, and plaque & media (P&M) (P&M = EEM—lumen) areas were obtained by manual contour detection. Percent atheroma volume was calculated as the proportion of the entire vessel wall occupied by atherosclerotic plaque throughout the segment of interest.

The following equation calculates the percent atheroma volume: Σ P&M area/ ΣEEM area X 100 ^4^; for eccentricity, plaque plus media eccentricity was evaluated.

Arc, location, and length of calcification were also evaluated. To assess the plaque vulnerability, the ratio of a slice with unconfirmed EEM (＞90°) to lesion length was evaluated due to backward attenuation in the absence of calcification in each image.

In OCT analysis, two expert investigators, blinded to the clinical presentation, performed offline image analysis; discordance was resolved by consensus. The corresponding arterial segment in initial images and images after pre-dilatation was identified by the guidance of anatomical landmarks and analyzed at similar intervals of 0.4 mm.

Flow and lumen area were measured to evaluate the degree of stenosis. In addition, characteristics (red or white), area (thrombus area, thrombus burden), volume (thrombus volume), and length of the thrombus were measured.

A semi-quantitative assessment was also performed using the OCT-thrombus score, with the application of a previously published method.^5,6^

For calcification, each calcium deposition in the target lesion was evaluated by three parameters: maximum angle, maximum thickness, and length; and a calcification score was calculated.^7^

The presence or absence of plaque rupture often could not be assessed from the initial image due to the severity of the thrombus and stenosis; therefore, it was evaluated with both initial and after balloon dilatation.

1. **Coronary CT angiogram analysis**

All patients in this study underwent cardiac CT assessment using a 320-row detector CT scanner (Aquilion One Vision; CANON Medical Systems Corp., Tokyo, Japan).　As described in the SCCT Guideline,^8,9^ the visual assessment was used for evaluation, and the restenosis was defined as lumen stenosis of more than 50% (Grade 3-5).

**Supplementary Tables**

**Table S1. Evaluation indices for each imaging device**

| Imaging indices | OCT  (N=14) | IVUS  (N=16) |
| --- | --- | --- |
| EEM | - | + |
| Lumen | + | + |
| Eccentricity | - | + |
| Plaque & media | - | + |
| Percent atheroma volume | - | + |
| Calcification | | |
| Arc | + | + |
| Location | - | + |
| Thickness | + | - |
| Length | + | + |
| Calcium score | + | - |
| Thrombus | | |
| Red or white | + | - |
| Area/Burden | + | - |
| Volume | + | - |
| Length | + | - |
| Thrombus score | + | - |
| Plaque rupture | + | - |
| EEM: external elastic membrane |  |  |

**Table S2. IVUS findings**

|  | Bailout stent  (N=2) | RYUSEI® DCB (N=12) | p-value |
| --- | --- | --- | --- |
| EEM area, mm^2^ | 13±1.3 | 14±7.4 | ns |
| Lumen, mm^2^ | 3.3±0.46 | 3.8±1.6 | ns |
| Lumen area stenosis, % | 82.3±10.3 | 77.7±9.5 | ns |
| minimal lumen area, mm^2^ | 1.48±0.33 | 1.6±0.6 | ns |
| plaque atheroma volume, mm^3^ | 73.2±0.85 | 67.7±8.7 | ns |
| plaque eccentricity index | 0.18±0.02 | 0.18±0.09 | ns |
| plaque & media eccentricity | 0.57±0.01 | 0.67±0.21 | ns |
| remodeling index | 1.00±0.02 | 0.72±0.26 | 0.08 |
| EEM error without calcification | 14% (9.5-19) | 11% (0-18.6) | ns |
| Calcification |  |  |  |
| arc: ave/max° | 74±53.2/225±134 | 65±42/164±79.4 | ns |
| location deep, (%) | 2(100) | 10(83) | 0.06 |
| Length, mm | 12±8.5 | 8.8±5.2 | ns |
| Calcium score | 24±14 | 17±9.2 | ns |
| The data are expressed as n (%), mean ± SD, or median (interquartile range).  EEM: external elastic membrane; ave: average; ns, non-significant | | | |

**Table S3.　CCTA evaluation of RYUSEI®DCB group**

| Pt No | Dx | Target | QCA  Final　Diameter stenosis | CCTA | | | | |
| --- | --- | --- | --- | --- | --- | --- | --- | --- |
|  |  |  |  | Agatston  score | | Diameter stenosis | | |
|  |  |  |  | Total | Target vessel | discharge | 1-year | 2-year |
| 1 | STEMI | 4PD | 26 | 80 | 33 | mild | minimal | visit refusal |
| 2 | NSTEMI | 2 | 14 | 1269 | 448 | mild | mild | mild |
| 4 | STEMI | 7 | 36 | 284 | 152 | minimal | mild | mild |
| 5 | STEMI | 1 | 32 | 614 | 210 | mild | mild | mild |
| 7 | STEMI | 9 | 32 | 394 | 238 | minimal | mild | minimal |
| 8 | STEMI | 13 | 21 | 78 | 26 | mild | mild | mild |
| 9 | Unstable angina | 1 | 49 | 701 | 230 | mild | moderate | moderate |
| 12 | Unstable angina | 15 | 36 | 370 | 25 | mild | mild | mild |
| 13 | STEMI | 1 | 47 | 6274 | 3015 | moderate | TLR case 1 | |
| 14 | STEMI | 7 | 15 | 176 | 4 | moderate | N/A for CKD | |
| 16 | STEMI | 6 | 49 | 1661 | 806 | moderate | severe | TLR case2 |
| 17 | NSTEMI | 4AV | 20 | NA | 847 | minimal | minimal | minimal |
| 18 | STEMI | 7 | 33 | 675 | 529 | N/A (artifact) | ※1 | minimal |
| 19 | STEMI | 3 | 17 | 372 | 160 | mild | minimal | minimal |
| 20 | NSTEMI | 1 | 16 | 94 | 17 | minimal | moderate | mild |
| 21 | STEMI | 1 | 37 | 0 | 0 | moderate | moderate | moderate |
| 22 | STEMI | 6 | 20 | 330 | 291 | minimal | minimal | visit refusal |
| 23 | STEMI | 1 | 30 | 1205 | 226 | moderate | minimal | normal |
| 24 | STEMI | 3 | 22 | 33 | 0 | moderate | visit refusal | visit refusal |
| 25 | NSTEMI | 6 | 37 | 32 | 26 | mild | minimal | mild |
| 27 | NSTEMI | 15 | 19 | 303 | 61 | mild | mild | ※2 |
| 28 | STEMI | 6 | 32 | 1 | 1 | minimal | mild | normal |
| 29 | STEMI | 3 | 37 | 190 | 64 | mild | visit refusal | visit refusal |
| 30 | STEMI | 7 | 18 | 693 | 605 | moderate | moderate | non-cardiac death |
| The data are expressed as n. STEMI: ST-segment elevation myocardial infarction; NSTEMI: Non ST-segment elevation myocardial infarction; PD: posterior descending branch; AV: atrioventricular branch; QCA; quantitative coronary angiography; CCTA: coronary computed tomography angiography; N/A: not available; ※1: CAG evaluation of % stenosis: 25%; ※2: CAG evaluation of % stenosis: 25%; TLR: Target Lesion Revascularization | | | | | | | | |

**References**

^1^ Raber L, Mintz GS, Koskinas KC, et al. Clinical use of intracoronary imaging. Part 1: guidance and optimization of coronary interventions. An expert consensus document of the European Association of Percutaneous Cardiovascular Interventions. Eur Heart J 2018;39(35):3281-300.

^2^ Saito Y, Kobayashi Y, Fujii K, et al. Clinical expert consensus document on standards for measurements and assessment of intravascular ultrasound from the Japanese Association of Cardiovascular Intervention and Therapeutics. Cardiovasc Interv Ther 2020;35(1):1-12.

^3^ Fujii K, Kubo T, Otake H, et al. Expert consensus statement for quantitative measurement and morphological assessment of optical coherence tomography. Cardiovasc Interv Ther 2020;35(1):13-8.

^4^ Nicholls SJ, Hsu A, Wolski K, et al. Intravascular ultrasound-derived measures of coronary atherosclerotic plaque burden and clinical outcome. J Am Coll Cardiol 2010;55(21):2399-407.

^5^ Kajander OA, Koistinen LS, Eskola M, et al. Feasibility and repeatability of optical coherence tomography measurements of pre-stent thrombus burden in patients with STEMI treated with primary PCI. Eur Heart J Cardiovasc Imaging 2015;16(1):96-107.

^6^ Amabile N, Hammas S, Fradi S, et al. Intra-coronary thrombus evolution during acute coronary syndrome: regression assessment by serial optical coherence tomography analyses. Eur Heart J Cardiovasc Imaging 2015;16(4):433-40.

^7^ Fujino A, Mintz GS, Matsumura M, et al. A new optical coherence tomography-based calcium scoring system to predict stent underexpansion. EuroIntervention 2018;13(18):e2182-e9.

^8^ Leipsic J, Abbara S, Achenbach S, et al. SCCT guidelines for the interpretation and reporting of coronary CT angiography: a report of the Society of Cardiovascular Computed Tomography Guidelines Committee. J Cardiovasc Comput Tomogr 2014;8(5):342-58.

^9^ Wu FZ, Wu MT. 2014 SCCT guidelines for the interpretation and reporting of coronary CT angiography: a report of the Society of Cardiovascular Computed Tomography Guidelines Committee. J Cardiovasc Comput Tomogr 2015;9(2):e3.
